# Pathophysiology, clinical manifestations and current management of IL-1 mediated monogenic systemic autoinflammatory diseases, a literature review

**DOI:** 10.1186/s12969-022-00728-0

**Published:** 2022-10-17

**Authors:** Yandie Li, Meiping Yu, Meiping Lu

**Affiliations:** Department of Rheumatology Immunology and Allergy, Children’s Hospital, Zhejiang University School of Medicine, National Clinical Research Center for Child Heath, NO.57 Zhugan Lane, Yan-an Road, Hangzhou, 310003 China

**Keywords:** Systemic autoinflammatory disease, IL-1, Pathophysiology, Clinical manifestations, Treatment

## Abstract

**Background:**

Systemic autoinflammatory diseases (SAIDs) are hyperinflammatory and immune-dysregulation conditions that present in childhood. This kind of disease is a rare disease with early-onset, severe condition and difficult diagnosis, which seriously affects the growth and development of children. Most children need a genetic diagnosis. However, with the limitation of access to genetic testing and the detection of somatic mutations, the diagnosis of SAIDs remains challenging. IL-1 is one of the important cytokines involved in the pathogenesis of SAIDs. Here we briefly review monogenic SAIDs mediated by aberrant IL-1 production, with the aim to further understand the pathogenesis, clinical manifestations and treatments of IL-1 mediated SAIDs.

**Methods:**

Literature reviews were performed using “PubMed” and “Web of Science” by searching for the terms “autoinflammatory diseases” and “IL-1”.

**Results:**

Monogenic SAIDs mediated by IL-1 include MKD, FMF, TRAPS, PAAND, PAPA, CAPS, DIRA, Majeed syndrome, NAIAD, NLRC4-MAS, PFIT, APLAID**.** Monogenic SAIDs have early onset, various clinical manifestations and difficult diagnosis, so early recognition and early treatment can reduce the complications and enhance the quality of life.

**Conclusions:**

There are many kinds of IL-1 mediated SAIDs. Pediatricians should be alert to SAIDs in the face of the patients with repeated fever, repeated rash and poor effect of routine treatment. The patients should be carried out with gene testing and treatment in time.

## Background

Systemic autoinflammatory diseases (SAIDs) are a group of conditions characterized by exaggerated innate immune system response, leading to sterile inflammation involving multiple organ systems. Gene mutation-induced aberrant production of cytokines result in SAIDs. Since Kastner first proposed the concept of SAIDs in 1999 [1], there have been over 40 syndromes classified into this category by the International Union of Immunological Societies [2]. Although sharing some common characteristics, it should be noted that SAIDs are different from autoimmune diseases and have different pathophysiologies. Mendelian SAIDs are a group of inherited diseases characterized by early-onset and systemic inflammation. They are caused by mutations in genes involved in the regulation of innate immune responses, resulting in over-production of inflammatory cytokines that fuel the inflammatory phenotype, but with lack of involvement of the adaptive immune system (absence of autoreactive T lymphocytes and autoantibodies). SAIDs that are onset in perinatal period are not common in clinic, but their symptoms overlap with infections and hematological malignancies, making their diagnosis more difficult. Therefore, there are still significant challenges in the early identification, early diagnosis and rational treatment of SAIDS.

A variety of cytokines are involved in the pathogenesis of monogenic SAIDs, among which IL-1-related SAIDs are more common. Due to the overlap of the pathogenesis of SAIDs, different scholars also have different classification methods for this kind of diseases. Here we review monogenic SAIDs mediated by IL-1, aiming at familiarizing pediatricians with SAIDs mediated by IL-1 and accelerate early diagnosis and treatment initiation [3].

Next, we will introduce the pathogenesis, clinical manifestations and current treatment measures of several SAIDs in detail (Table 1 and Table 2).

**Table 1:** Summary of the gene, transmission and clinical manifestations of IL-1 mediated monogenic SAIDs

SAIDS	Gene (encode protein)	Transmission	clinical manifestation
MKD	*MVK* (mevalonate kinase)	AR	
● HIDS	Mild		high fever, rash, gastrointestinal symptoms, lymphadenopathy, splenomegaly, joint pain, and even amyloidosis
● MA	Severe		typical HIDS characteristics, mental retardation, dysplasia and mental retardation
FMF	*MEFV* (pyrin)	AR	periodic fever, abdominal pain, chest pain, serositis, rash, amyloidosis
TRAPS	*TNFRSF1A* (TNF receptor type 1)	AD	long term periodic fever, abdominal pain, periorbital edema
PAAND	*MEFV* (pyrin)	AD	fever, pustular acne, pyoderma, neutrophilic dermatosis, arthralgia
PAPA	*PSTPIP1* (PSTPIP1)	AD	pyoderma, suppurative arthritis, severe cystic acne
CAPS	*NLRP3/CIAS1* (cryoporin)	AD/de novo mutation	
● FCAS	Mild		cold-induced urticaria, fever, arthralgia
● MWS	Moderate		fever, rash, arthralgia, sensorineural deafness
● NOMID	Severe		fever, rash, arthritis, aseptic deafness meningitis and sensorineural in newborns
DIRA	*IL1RN* (IL-1 receptor antagonist)	AR	osteitis, pustular lesions
Majeed syndrome	*LPIN2* (LIPIN2)	AR	anemia, osteomyelitis, neutrophilic dermatosis
NAIAD	*NLRP1* (NLRP1)	AD/AR	systemic inflammation, arthritis, dyskeratosis
NLRC4-MAS	*NLRC4* (NLRC4)	AD	repeated MAS, enteritis, cold-induced fever and urticaria, central nervous system inflammation
PFIT	*WDR1* (WDR1)	AR	periodic fever, immune deficiency, thrombocytopenia
APIAID	*PLCG2* (phospholipase Cg2)	AD	cold urticaria, pulmonary interstitial lesions, recurrent blisters, arthralgia, ocular inflammation, enterocolitis and antibody deficiency

**Table 2:** Summary of the current management of IL-1 mediated SAIDs

SAIDs	current management	Recommendation Index
MKD	canakinumab	Recommendation A, approved by the FDA, strong evidence for the effectiveness [10, 11]
	anakinra	Recommendation B, canakinumab is unavailable [9]
	tocilizumab	Recommendation C, IL-1 blockade fails or is unavailable [13]
	etanercept	Recommendation C, IL-1 blockade fails or is unavailable [12]
	NSAIDS or glucocorticoids	Recommendation D, mildly affected patients
	allogeneic stem cell transplantation	Severest cases [17]
	hemodialysis, kidney transplantation	AA-amyloidosis, kidney failure [15]
FMF	Colchicine	Recommendation A, early use after diagnosis [20]
	canakinumab	Recommendation B: the second line: colchicine resistance or intolerance to colchicine, approved by the FDA [23]
	anakinra	Recommendation C [22]
TRAPS	canakinumab	Recommendation A, approved by the FDA [31]
	NSAIDS or glucocorticoids	Mildly affected patients [27]
	etanercept	Case report [28]
	anakinra	Case report [29]
	tocilizumab	Case report [30]
	hemodialysis, kidney transplantation	AA-amyloidosis, kidney failure [34]
PAAND	anakinra	Systemic and/or gastrointestinal inflammation [35]
	adalimumab	Cutaneous and/or articular manifestations [35]
	isotretinoin	Cutaneous component [35]
	canakinumab	Systemic and/or gastrointestinal inflammation [35]
	colchicine and low-dose prednisolone	Case report [36]
PAPA	NSAIDS or glucocorticoids	Mildly affected patients [41]
	etanercept, adalimumab and infliximab	Case report [42]
	anakinra	Case report [41]
	Canakinumab	Case report [43]
	retinoids	Case report [41]
CAPS	anakinra	Approved by the FDA and EMA [49]
	canakinumab	Approved by the FDA and EMA [52]
	rilonacept	Approved by the FDA [51]
	NSAIDS or glucocorticoids	Combination with biological agents [53, 54]
	tocilizumab	Case report [55]
DIRA	anakinra	Case report [60, 61]
	canakinumab	Case report [62]
	rilonacept	Case report [61]
Majeed syndrome	NSAIDS or glucocorticoids	The first-line [70]
	anakinra	Case report [67]
	canakinumab	Case report [67]
	TNF-α blockers or bisphosphonates	Refractory cases [70]
NAIAD	corticosteroids	Case report [72]
	acitretin	Case report [71]
	etanercept or anakinra	Case report [71]
NLRC4-MAS	IL-1 blockade	The first-line [74]
PFIT	hematopoietic stem cell transplantation	Case report [78]
APIAID	Glucocorticoids	Case report [79]
	IVIG	Case report [79]
	adalimumab	Case report [79]
	IL-1 blockers	Case report [84]

### MKD (Mevalonate Kinase Deficiency)

MKD is an autosomal recessive (AR) disorder caused by loss-of-function (LOF) mutations in mevalonate kinase (*MVK*), an enzyme in the cholesterol biosynthesis pathway. The mutated *MVK* has approximately only 10% residual enzyme function. Mutations in *MVK* lead to decreased production of nonsterol isoprenoids, which activates the pyrin inflammasome, resulting in caspase-1 mediated IL-1β release [4]. The disorder encompasses two phenotypes: hyperimmunoglobulinemia D and periodic fever syndrome (HIDS) and mevalonic aciduria (MA). Whereas a partial deficiency in *MVK* causes the milder HIDS phenotype, the nearly absent enzymatic activity causes the more severe form of MKD, MA. HIDS manifests as high fever, rash, gastrointestinal symptoms, lymphadenopathy, splenomegaly, arthralgia and pharyngitis. A small percentage of patients develop AA amyloidosis (AA) with kidney dysfunction. Patients with MA have not only typical characteristics of HIDS, but also psychomotor retardation, dysmorphic features, and failure to thrive [5]. Lesional skin biopsies show endothelial cell swelling, fibrinoid necrosis of vessel walls, and a perivascular neutrophilic and lymphocytic infiltrate. Perivascular deposits of IgD and C3 in a granular staining pattern are found in skin samples from some patient [6]. By the end of 2018, 300 MKD patients have been reported worldwide, which is likely an underestimation of the actual number given the lack of genetic screening programs and knowledge of this disease worldwide. The predominant variants including p.A148T and p.V377I present in MKD patients in the Dutch population. The p.V377I variant is the most common in patients with MKD. And in the healthy Dutch population, the allele mutation frequency of p.V377I was as high as 0.003 [7]. This relatively high carrier rate would imply a much higher disease incidence than what is typically reported and suggests that homozygotes for the V377I variant might exhibit a much milder phenotype of MKD or no disease phenotype in individuals.

On-demand use of nonsteroidal anti-inflammatory drugs (NSAIDs) and corticosteroids relieve or partially improve symptoms and may be sufficient in patients with milder symptoms. However, in most patients, these drugs do not prevent the symptoms. In recent years, more and more children receive biological medicines, including anti-tumor necrosis factor-alpha (TNF-α) agents (infliximab, etanercept, and adalimumab), anti-IL1 agents anakinra (recombinant anti-IL1 receptor antagonist), and canakinumab (a human anti-IL-1β monoclonal antibody), and tocilizumab(a monoclonal antibody targeted against the IL-6 receptor) [8].

Since IL-1β plays an important role in the inflammatory phenotype of MKD, anakinra and canakinumab have been employed in a substantial number of patients. A prospective observational study including 11 MKD patients described the usage of anakinra. Patients with a more severe phenotype were assigned to continuous treatment, and patients with a milder phenotype were free to choose between constant or on-demand treatment (children < 16 years received 1.6 mg/kg/day, to be raised to a maximum of 2 mg/kg/day; adults received 100 mg/day, to be raised to a maximum of 200 mg/day in cases of failure). This study showed that treatment was accompanied by shortening of fever time, lower c-reactive protein (CRP) levels and reduction of symptoms, but not lead to reduced frequency of fever episodes. Adverse events included local injection site reactions and upper respiratory tract infections (*n* = 2) [9].

Although anakinra has been beneficial for many patients, the long-term acting canakinumab has been far better studied. Canakinumab is more well-studied and effective treatment for MKD patients [10]. In a large, randomized controlled trial, they investigated the efficacy of canakinumab in periodic fever patients, including 72 MKD patients. This study showed that treatment with canakinumab, 150 mg (or 2 mg/kg in children < 40 kg) every 4 weeks, led to complete remission in 35% of patients after 16 weeks, compared to 6% in patients receiving placebo. A dose increase to 300 mg (or 4 mg/kg in children < 40 kg) every 4 weeks further increased the effect to 57%. In a minority of patients, the dose could be reduced to 150 mg (or 2 mg/kg in children < 40 kg) every 8 weeks. Patients who did not achieve complete remission still had a significant reduction in attack frequency. Seven serious adverse events were reported while using canakinumab. For the current study, canakinumab is the first choice for the treatment of MKD [11].

Etanercept has been beneficial in a number of MKD patients. Etanercept had been described in a retrospective case series in 27 MKD patients, with 16 patients reporting some benefit and 11 patients not responding. Two of these 16 patients reached complete remission. Another retrospective study described 8 MKD patients assigned to etanercept, with 7 achieving complete remission. Given the evidence for IL-1 blockade, etanercept is not a first-choice option but is mainly used to treat patients who have no access to or no response to IL-1 blockade [12].

Tocilizumab is not widely used to treat patients with MKD. But, a 12-year-old girl with MA who was resistant to IL-1 and TNF-α blockade commenced intravenous tocilizumab (8 mg/kg every 2 weeks). She was in complete clinical and serological remission after the fifth dose [13]. A study reported a 4-year-old girl presenting with recurrent episodes of fever, arthritis, and gastroenteritis since 3 months of age, who was diagnosed with MKD and was prescribed naproxen and oral prednisolone at 1 mg/kg/d and isoniazid prophylaxis for latent tuberculosis. However, the symptoms flared on tapering steroids, then tocilizumab at 12 mg/kg four-weekly was started, which resulted in remission of symptoms [14].

The disease can lead to AA and ultimately kidney failure. The Eurofever cohort reported five patients with AA (in a cohort of 114 patients). Four of these patients underwent kidney transplantation due to end-stage kidney failure, while one patient died due to the complications of dialysis. Therapy with biologicals could be kept on before kidney transplantation when patients underwent hemodialysis [15]. A 3-year-old with MA was not respond to treatment with anakinra and etanercept and then allogeneic bone-marrow transplantation from an HLA-identical sister was performed. Follow-up after 15 months showed complete remission with normal inflammatory parameters, although the patients still had persistent atactic gait [16]. However, a Turkish boy with severe MA led to ascites and respiratory distress. After treatment with glucocorticoids and canakinumab had failed, an allogeneic bone marrow transplantation from an HLA-identical sister was performed [17].

### FMF (Familial Mediterranean Fever)

FMF is the earliest described and most prevalent SAID, first described in 1945, with more than 100,000 affected individuals around the world [18]. FMF is commonly seen in people of Mediterranean and Middle Eastern descent, including Jews, Armenians, Arabs, Kurds, Greeks, Turks, Iranians, and Italians. The disease was caused by gain-of-function (GOF) mutations in *MEFV* (encoding the protein pyrin), which is located on the short arm of chromosome 16 (16 p13.3). And the disease is predominately recessive, with heterozygous carriers occurring at frequencies as high as 1:4 in certain populations. Pyrin is predominantly expressed in neutrophils, which are the main effectors or cells associated with inflammation in FMF. Although the role of pyrin in IL-1 activation remains disputed, one hypothesis is that it suppresses the activation of pro-caspase-1, thereby inhibiting inflammasome activation. Thus, defective pyrin in FMF may exhibit a less inhibitory effect on the inflammasome, leading to unchecked production of IL-1β. Poorly controlled inflammation accumulates and depletes serum amyloid A in the kidney, leading to the most dreaded complication, renal failure. Lesional biopsies show dermal edema with a perivascular and interstitial dermal infiltrate composed of neutrophils and lymphocytes. Mild hyperkeratosis and acanthosis can be seen in the epidermis [19]. FMF is characterized by the periodic fever of 3–4 days duration, serositis presenting as abdominal or pleuritic chest pain, mono-arthritis (hip, knee, or ankle), and elevation of acute phase reactants [such as ESR (erythrocyte sedimentation rate), CRP, haptoglobin, fibrinogen)] [20]. However, the diagnosis of FMF is sometimes tricky in patients with atypical symptoms and features. Patients can present with asymptomatic proteinuria, while sometimes nephrotic syndrome or end-stage kidney disease can be the first manifestation of FMF. Male sex and Mediterranean origin are risk factors for developing amyloidosis [21].

Colchicine remains the primary treatment for FMF patients [20]. It decreases the attacks, improves the quality of life, prevents amyloidosis, and should be started once the clinical diagnosis is established. Most FMF patients do very well on adequate doses of colchicine. The treatment with colchicine is usually lifelong. Dosing varies according to age and severity of symptoms. Children under five years can be given 0.03–0.07 mg/kg/day of colchicine. 1 mg colchicine is ideal for children over ten years and adults. Patients who undergo renal transplantation due to amyloidosis-related end-stage renal disease should continue receiving colchicine. If a maximum dose of colchicine (up to 3 mg in adults) does not improve the frequency and severity of the attacks, then colchicine resistance should be suspected.

IL-1 inhibitors are the second-line drugs for patients who have colchicine-resistant FMF or who have an intolerance to colchicine. The M694V homozygous FMF patients tend to be more resistant of colchicine, who should receive anakinra or canakinumab. In colchicine-resistant FMF patients, anakinra had shown to be effective and safe in a randomized clinical trial including 25 patients. The beneficial effect was observed in the number of attacks per month, remission of nephrotic syndrome, and improved quality of life [22]. Canakinumab use led to a complete response in colchicine resistant FMF in about 67.5%, partial response in the remaining 32.5%, and no reports of treatment failure in a recent systematic review of eight studies including 40 patients. It also decreased urine protein in one patient with type AA. There were no reported serious side effects while on canakinumab that warranted discontinuation. Canakinumab is the only biologic agent approved by the Food and Drug Administration (FDA) to treat colchicine-resistant FMF [23]. In some cases, TNF-α inhibitors infliximab, and etanercept have also been described, but the actual efficacy is still not established.

### TRAPS (Tumor necrosis factor Receptor-Associated Periodic fever Syndrome)

TRAPS was initially described in 1982 in an Irish family with 16 affected members over three generations. TRAPS is an autosomal dominant (AD) disorder due to heterozygous mutations in the TNF receptor superfamily 1A (*TNFRSF1A*) gene on chromosome 12, which encodes for the TNF-α receptor. Most cases are reported in Caucasian and Asian populations. In TRAPS, accumulation of mutant TNFR1 protein within the endoplasmic reticulum and abnormal autophagy causes IL-1β production [24]. In this review, we classify TRAPS as IL-1 mediated monogenic SAIDs. TRAPS is also known as protein folding disorder in other literatures. The syndrome presents clinically with acute inflammatory episodes of 1–3 weeks. These conditions are characterized by unprovoked episodic sterile inflammation manifesting as unexplained fevers, skin rashes, serositis, arthralgia and myalgia, migratory erythematous rash, and eye inflammation. Still, the symptoms of central nervous system are relatively rare in TRAPS [25]. These episodes usually resolve spontaneously and are not associated with the production of autoantibodies or autoreactive T cells. During febrile episodes, patients have elevated acute phase reactants. Some can have extremely high levels of serum amyloid A (SAA), predisposing them to AA. AA is the most severe complication of TRAPS. Lesional skin biopsies show a mild perivascular lymphocytic infiltrate in the edematous areas of the papillary dermis. C3 and C4 are deposited in the perivascular of the dermis [26].

Short-term corticosteroids and NSAIDs help abort attacks and provide symptomatic relief during episodes. However, prolonged treatment and escalating doses are required to control chronic TRAPS symptoms. Long-term therapy with corticosteroids was ineffective in preventing the development of amyloidosis or reducing the frequency or intensity of inflammatory attacks.

In an open-label study of patients with TRAPS, etanercept also reduced the levels of acute-phase reactants during asymptomatic periods and decreased the use of NSAIDs and glucocorticoids [27]. Treatment with etanercept might be efficacious in preventing AA in some patients [28]. Adalimumab (a full humanized anti-TNF monoclonal antibody) and infliximab (a chimeric mouse-human monoclonal antibody) were reported to cause worsening of disease flare in TRAPS patients.

Anakinra induced a complete response in 79% of TRAPS patients and a partial response in 16% [29]. Macrophage-activation syndrome (MAS) was described in an 11-year-old girl with TRAPS, who treated with tocilizumab to improve symptoms and prevent attacks rapidly [30].

Canakinumab was approved by the FDA for TRAPS in 2013. Patients with TRAPS were treated with canakinumab, all clinical symptoms were utterly relieved, and inflammatory markers were normalized [31]. Gattorno et al. evaluated the efficacy of canakinumab in 20 patients with TRAPS in phase II clinical trial over 4 months, followed by a 5-month withdrawal period, and with the reintroduction of canakinumab on disease flare for long-term treatment for another 24 months. The primary endpoint was clinical remission and complete or partial serological remission on day 15. Most patients (95%) reached the primary endpoint. After stopping canakinumab, all patients relapsed with a median recurrence time of 91.5 days. After reintroducing canakinumab, all patients underwent clinical and serological remission [32]. In a phase III randomized, double-blind placebo-controlled study, 46 TRAPS patients were followed for a 12-week screening period and then randomized at the flare onset to a 16-week treatment period with 150 mg every four weeks for canakinumab or placebo. The proportion of responders at the end of week 16 was 45% (10/22 patients) in the treatment group and 8% (2/24 patients) in the placebo group. During the withdrawal period, where the canakinumab was administered over a prolonged dosing interval (8 weeks), 53% of the patients maintained controlled disease. Adverse reactions were mild and consisted of mild infections and injection site reactions [33].

Delaleu et al. reported that 41 TRAPS with AA. AA diagnosis preceded that of TRAPS in 96% of cases, and 47% required renal replacement therapy [34].

To sum up, biological strategies that block specific immune mediators such as TNF-α or IL-1 are effective in suppressing TRAPS symptoms, preventing reactive amyloidosis, and halting the progression to organ damage.

### PAAND (Pyrin-Associated Autoinflammation with Neutrophilic Dermatosis)

PAAND is another disorder also driven by mutations in the *MEFV* gene and follows a dominant inheritance pattern. PAAND was first reported in 2016. However, PAAND presents with distinct clinical manifestations that differ from FMF. The pyrin mutation S242R abolishes the ability of pyrin to bind to 14–3-3 as 14–3-3 binding is essential for pyrin’s autoinhibited conformation, mutation of the binding motif results in activation of the inflammasome. A family exhibiting PAAND-like features harbored an E244K substitution in the + 2 position of a 14–3-3 binding motif in pyrin, again disrupting 14–3-3 binding. Both S242R and E224K pyrin induce spontaneous inflammasome formation [35]. Although heterozygous mutations initially reported PAAND, a recent homozygous Serine-208 mutation was described. Excessive IL-1β and activation of inflammatory pathways cause a clinical picture characterized by neutrophilic dermatosis, recurrent fever, increased acute-phase reactants, myositis, myalgia and arthralgia [28]. Furthermore, increased levels of cleaved caspase-1 and IL-1β in skin biopsies from PAAND patients provided the rationale for initiating treatment targeting IL-1β.

Given the novelty of the disorder and the lack of case reports, evidence regarding therapeutic options is currently limited. Two siblings, a girl (proband; age 5 years) and a boy (age 2.5 years) of Iranian-Azeri ancestry, were born to first-cousin consanguineous parents. The clinical features were recurrent episodes of maculopapular and pustular rash and gastrointestinal involvement resembling inflammatory bowel disease (IBD). A trio-WES test detected a homozygous missense variation, p.Ser242Gly, in both patients' *MEFV* genes. Under the guidance of the *MEFV* genotype, treatment with colchicine (1 mg/d) and low-dose prednisolone (2.5 mg every other day) was started, and the patients responded well. This study demonstrated successful genotype-guided treatment with colchicine and low-dose prednisolone in patients with PAAND, a low-cost therapeutic option with minimal adverse effects [36].

Anakinra treatment has been successful in most PAAND patients. A case of PAAND was refractory to treatment with anakinra, whereas adalimumab (anti-TNF-α) reached an immediate and sustained clinical response [35].Treatment of PAAND patients with anakinra did not prove superior to therapy with anti-TNF-α agents. In PAAND, gastrointestinal symptoms were better controlled with anakinra. We would opt for treatment with anti-TNF-α in case of predominant and severe cutaneous and/or articular manifestation. The cutaneous component has responded well to isotretinoin. IL-1 blockade can be proposed when the clinical phenotype comprises mainly systemic and/or gastrointestinal inflammation [35].

### PAPA [Pyogenic arthritis, pyoderma gangrenosum (PG) and acne]

Pyogenic arthritis, pyoderma gangrenosum (PG) and acne (PAPA) syndrome were first reported in 1997. It is an autosomal dominant autoinflammatory disease due to mutations in proline-serine-threonine phosphatase interacting protein 1 (*PSTPIP1*) gene and presenting with cutaneous and articular manifestations [37]. Painful, recurrent, sterile monoarticular arthritis with a prominent neutrophilic infiltrate usually occurs in childhood and may be the presenting sign of the disease. Since pyoderma and cystic acne may not appear in the early course of disease, PAPA syndrome is prone to be misdiagnosed as a joint abscess.

PAPA syndrome has been recently included in the group of PAPA spectrum disorders. PAPA is the most common type of PAPA spectrum disorder [38]. *PSTPIP1* has a role in innate immunity and several inflammatory pathways. It interacts with pyrin, encoded by the *MEFV*, which is found in association with the cytoskeleton in monocytic cells and modulates immunoregulatory functions. Pyrin and *PSTPIP1* form homotrimers, but in this unbound conformation, the PYD (protein pyrin domain) of pyrin is masked by its B box. The binding of *PSTPIP1* to the pyrin’s B box unmasks the PYD and this event leads to interaction and dimerization of ASC (Apoptosis-associated speck-like protein containing a CARD) [39]. Consequently, the formation of the ASC pyroptosome recruits and activates caspase-1 and IL-1β production. Its complex pathophysiology includes disordered keratinization with abnormal sebaceous stem cell differentiation. It has recently been demonstrated that an autoinflammatory component induced by Propionibacterium acnes via inflammasome activation is also involved, thus linking acne to classic SAIDs. As expected, increased amounts of IL-1β and TNF-α have been detected in peripheral blood mononuclear cells of PAPA patients and augmented levels of IL-18 have been observed in the serum of these patients [40].

A total of 76 patients with PAPA syndrome reported in 29 articles were included in Wang’s literature review [41]. The classical triad of arthritis, PG, and acne was visible in only 16 (25.4%) patients, while 24 (38.1%) exhibited only one major symptom. Skin lesions were more commonly seen in patients with adult-onset disease than those with childhood-onset disease (100% vs 83%), whereas arthritis was less common (50% vs. 98.1%). Steroid and/or biological agents were effective in most patients [41].

There are no standardized treatments for PAPA syndrome because of the rarity of the disease. Most evidences derive from case reports. The first cases of PAPA reported were treated with systemic or intraarticular corticosteroids, nonsteroidal anti-inflammatory drugs or antibiotics. Drugs directed against IL-1 and TNF-α have been reported to be useful in controlling the manifestations of PAPA syndrome. The anti-TNF-α antagonists etanercept, adalimumab and infliximab were demonstrated to be effective in several case reports. Anakinra was effective in managing arthritis in PAPA patients. Subsequently, its effectiveness was also confirmed also for cutaneous symptoms [42]. Recently, anakinra is allowed to treat osteolytic lesions in a patient with PAPA and severe arthritis. Canakinumab at a dosage of 150 mg every 8 weeks led to complete healing of PG and acne in a patient with PAPA-like syndrome [43]. Joint disease is also responsive to corticosteroids, but they may exacerbate acne. Joint effusions may also be surgically managed by means of drainage and/or intra-articular corticosteroid injections. Topical and systemic retinoids have been shown to be effective in the management of the most severe forms of acne [41].

### CAPS (Cryopyrin-Associated Periodic Syndromes)

Heterozygous mutations in gene NLRP3 lead to CAPS, which can manifest with cold-induced urticaria, fever, central nervous system inflammation and bone overgrowth. Mild papillary dermal edema and dilatation of superficial dermal capillaries can be found in lesional skin biopsy. Predominantly neutrophilic, peri-eccrine, and perivascular infiltrates are noted [44]. Features of vasculopathy or vasculitis are absent. CAPS includes three autoinflammatory syndromes, familial cold autoinflammatory syndrome (FCAS), Muckle-Wells syndrome (MWS) and neonatal-onset multisystem inflammatory disease (NOMID)/ Chronic infantile neurologic, cutaneous, articular (CINCA) syndrome [45]. These three disorders form a disease-severity spectrum, with FCAS on the milder end and NOMID on the severe end. Urticaria in FCAS is characterized by cold-induced episodes. It presents several hours after cold exposure and can last up to 12 h. In MWS, urticaria may persist for longer than those in FCAS. Progressive sensorineural hearing loss, coxitis, and osteitis leading to limb pain are characteristic features of MWS. Over 25% of patients develop amyloidosis, which can be fatal. Compared to FCAS and MWS, earlier urticaria-like eruptions are seen in NOMID/CINCA patients. A majority of NOMID/CINCA patients present with urticaria-like eruptions at birth, and almost all develop these lesions by 6 months of age. Urticarial lesions in NOMID/CINCA tend to be persistent. Arthropathy, hearing loss, and dysmorphic features including midface hypoplasia and frontal bossing, are common manifestations. Central nervous system inflammation is often the most devastating feature and can lead to chronic aseptic meningitis, cognitive impairment, and epilepsy [46].

Gain-of-function mutations in NLRP3 induce inflammasome activation, leading to excessive secretion of IL-1β in CAPS. Specific innate immune sensors such as NLRP3 have the capacity to form an oligomeric complex with pro-caspase-1, facilitated by the adaptor protein ASC. This results in the cleavage and activation of pro-caspase-1; subsequently, caspase-1 cleaves the precursor pro-inflammatory cytokines pro-IL-1β and pro-IL-18, mediating the release of their active forms resulting in pyroptosis. About 50–80% of CAPS patients have germline NLRP3 mutations. In those without germline mutation, somatic mutation with NLRP3 disease-causing mutations with mosaicism of only 4.5%-25% of transcripts can cause severe NOMID/CAPS. From inheritance, mutations in FCAS are mostly familial, whereas most NOMID patients have de novo mutations in NLRP3 [47].

Early diagnosis of CAPS may lead to early and successful treatment with anti-IL-1 medications. A number of studies on these disorders have demonstrated remarkable responses to IL-1 blockers, and IL-1 inhibition is recommended for the CAPS at any age because of excessive secretion of IL-1β. IL-1 inhibition can partially halt organ damage and should be initiated early on in the disease course. Eskola et al. discussed 3 cases of MWS, with good responses to biological agents like canakinumab, rilonacept, and anakinra [48].

Anakinra is administered daily subcutaneously and blocks the binding of IL-1α and IL-1β to the IL-1 receptor, proven to have long-term efficacy and safety in several studies. In a survey of 43 CAPS patients treated with anakinra for up to 5 years, the most reported serious adverse events were pneumonia and gastroenteritis [49]. Anakinra has been approved by the European Medicines Agency (EMA) and the FDA for CAPS. Anakinra has been recommended to be used in a dose of 1–8 mg/kg daily. Dosing depends on the severity of symptoms and can be up-titrated. The typical dosing regimen varies from 1 to 2 mg/kg/day for patients with FCAS, up to 10 mg/kg/day for critically ill patients with NOMID/CINCA. The CNS penetrance of anakinra seems to be superior, and therefore this might be the treatment of choice in cases with aseptic meningitis [50]. But the efficacy of this drug in controlling chronic complications like systemic amyloidosis and deforming arthritis is ambiguous. Headache, pyrexia and respiratory infections have been described as common side effects of anakinra therapy. Infections (pneumonia and gastroenteritis) and MAS are serious but rare side effects. These side effects were common in children < 2 years of age.

The recombinant soluble IL-1 receptor rilonacept binds to IL-1α and IL-1β. Weekly subcutaneous administration has shown a good safety and efficacy profile against CAPS. So far, rilonacept has only been approved by the FDA. The dose of rilonacept for adults is 160 mg/week and varied from 2.2 to 4.4 mg/kg/week in children [51].

Canakinumab is administered subcutaneously every four to eight weeks. Several studies have confirmed the long-term efficacy and safety of canakinumab against CAPS [52]. In patients with mild to moderate CAPS, 150 mg of canakinumab can be administered if the body weight is > 40 kg, or it can be dosed with 2 mg/kg for patients from 15 to 40 kg every four to eight weeks. For children (15–40 kg) with an inadequate response, a dose increase, up to 3–4 mg/kg, might be necessary, and dosing up to 8 mg/kg every four weeks has been described for NOMID/CINCA patients. Canakinumab has been approved by the EMA and FDA. But the effectiveness of canakinumab in real life may be significantly lower than that in clinical trials. A study in 2016 showed a complete response rate of 72% in clinical use versus up to 97% reported in clinical trials. Some CAPS patients on canakinumab have reported Vertigo and angle-closure glaucoma [33].

NSAIDs and systemic corticosteroids can be used for symptomatic relief during episodes. However, NSAIDs should not be used without concomitant IL-1 inhibition due to insufficient evidence that they will prevent organ damage if used alone [53, 54]. Biological therapy in CAPS not only leads to the control of acute symptoms but also prevents long-term complications. Besides, a Korean 4-year-old boy who presented with recurrent urticaria, arthralgia, and fever was diagnosed with juvenile idiopathic arthritis. He was treated with steroid and methotrexate,but the symptoms continued. Administration of tocilizumab improved all of his symptoms. Genetic testing shows finally, a p.Val72Met mutation was found in exon 1 of the NLRP3 gene in this patient, so he was diagnosed with CAPS [55]. Tocilizumab could be an important addition to the treatment of CAPS.

### DIRA (Deficiency of IL-1 Receptor Antagonist)

Deficiency of the IL-1 receptor antagonist (DIRA) was first described in 2009 and so far 20 patients have been reported [56]. LOF mutations in the *IL1RN* gene have been implicated in DIRA, a rare autosomal recessive condition in which unopposed signaling of the pro-inflammatory cytokines IL-1α and IL-1β through the IL-1 receptor leads to severe systemic inflammation. IL-1 receptor antagonist (IL-1Ra) is structurally similar to IL-1 but devoid of biological activity; it competes for occupancy of the IL-1 receptor. DIRA patients present systemic inflammation, pustular rashes, oral mucosal lesions, and elevation of acute phase reactants [56]. Multifocal periostitis, bony overgrowth and sterile painful osteomyelitis can be a sequela. Failure to recognize the disease and treat it without anakinra and canakinumab can lead to the development of a severe inflammatory response syndrome and death from multi-organ failure. Lesional skin biopsies show dense myeloperoxidase positive neutrophilic infiltrates in the epidermis and superficial dermis, formation of pustules around the hair shaft, acanthosis, and hyperkeratosis. Deep connective tissue can show vasculitis and perivascular neutrophilic infiltration [57]. Mortality is approximately 30% in early infancy. In a 12-year-old patient, complete remission was achieved following administration of canakinumab every 6 weeks [58]. A retrospective case series and clinical service description showed that 19/20 (95%) of pediatric patients achieved minimally active clinical disease activity with canakinumab monotherapy, and canakinumab was well tolerated, with only one child developing an infection requiring hospitalization during the study. These studies suggested that these IL-1 antagonists may be effective treatment, but the FDA has not approved these biological agents to treat DIRA [59].

Several case reports have described the rapid clinical improvement and complete recovery following treatment with anakinra in DIRA. A 5-month-old Indian girl presented at the third week of life with irritability, sterile multifocal osteomyelitis including ribs and clavicles, a mild pustular rash, and elevated acute phase reactants. PCR and Sanger sequencing confirmed a homozygous 22,216 bp deletion that spans the first four exons of *IL1RN*. Due to clinical suspicion of DIRA, anakinra was initiated, resulting in an anaphylactic reaction that triggered desensitization with subsequent marked and sustained clinical and laboratory improvement [60].

In an open-label pilot study, six DIRA patients (children, age 3–6 years), treated with daily anakinra, were treated with subcutaneous rilonacept for 24 months. A loading dose (4.4 mg/kg) was administered, followed by once-weekly injections (2.2 mg/kg) for 12 months. Dose escalation (4.4 mg/kg) was allowed if inflammatory remission was not maintained. Subjects in remission at 12 months continued rilonacept for an additional 12 months. Five patients increase their dose because of micropustules. After dose escalation, all patients were in remission on weekly rilonacept administration. All children are growing at normal rates and have normal heights and weights. Quality of life improved and no serious adverse events were reported [61]. Anakinra and rilonacept have been declared safe and efficient, whereas canakinumab has been reported in a single case only [62].

### Majeed syndrome

Majeed syndrome (MJS) was first described in 1989 as a rare autoinflammatory disease [63]. The syndrome is caused by autosomal recessive LOF mutations in *LPIN2*, which encodes the phosphatidic acid phosphatase LIPIN2 that catalyzes the conversion of phosphatidic acid to diacylglycerol in the endoplasmic reticulum membrane, a key step in lipid metabolism. Mechanistically, impaired lipin-2 function could increase inflammasome activity and therefore increase IL-1 production through a failure to preserve the proper lipid environment [64]. *L*ipin-2 has been shown to regulate the activation of the NLRP3 inflammasome by modulating P2X7 receptor activation.

MJS presents with early-onset recurrent osteitis and multifocal osteomyelitis, and congenital dyserythropoietic anemia. A study in India showed that patients with MJS also had other unreported symptoms, including abdominal pain, recurrent diarrhea and ear secretions [65]. Lesional skin biopsies show a dermal neutrophilic infiltrate with edema of the upper dermis without histologic evidence of vasculitis. Studies in lipin-2–deficient mice suggested that mice developed anemia but no osteomyelitis, which showed that MJS resulted from environmental and genetic factors [66].

Magnetic resonance imaging (MRI) is the gold standard radiological tool for diagnosis. In the absence of validated diagnostic criteria, MJS remains an exclusion diagnosis. Bone biopsy was not specific, but it may be necessary in unifocal or atypical cases to differentiate it from malignancy or infection.

Herlin et al. reported elevated levels of proinflammatory cytokines, including IL-1β, IL-6, IL-8, and TNF-α, in 2 patients with MJS during active disease. Anakinra and canakinumab had been used successfully in patients with MJS and resulted in drastic clinical and laboratory improvement, especially after the failure of TNF inhibitors [67]. Remission on IL-1 blocking treatment confirms the important role of IL-1 in MJS [68]. Other treatments such as bone marrow transplants or gene therapy have not yet been tried in MJS [69]. First-line treatments are NSAIDs and oral glucocorticoids, while bisphosphonates or TNF-α blockers can be used in refractory cases [70].

### NAIAD (NLRP1-associated autoinflammation with arthritis and dyskeratosis syndrome)

The NLRP1-associated autoinflammation with arthritis and dyskeratosis syndrome (NAIAD) is a novel monogenic autoinflammatory disease characterized by skin lesions (follicular hyperkeratosis), arthritis, recurrent fever of 3–4 days, and elevated acute phase reactants. This disorder was first reported in 2016. The molecular screening revealed a non-synonymous homozygous mutation in *NLRP1* (c.2176C > T; p.Arg726Trp) in two cousins born from related parents originating from Algeria and a de novo heterozygous mutation (c.3641C > G, p.Pro1214Arg) in a girl of Dutch origin. The three patients showed elevated systemic levels of caspase-1 and IL-18, which suggested the involvement of NLRP1 inflammasome. NLRP1 inflammasome is composed of NLRP1 (also known as NALP1), ASC, caspase-1, and caspase-5, which is similar to the NLRP3 inflammasome; assembly results in the activation of caspase-1 and processing and secretion of IL-1β. Both autosomal recessive and autosomal dominant inheritance have been described in NAIAD. Arthritis was treated with corticosteroids acitretin and etanercept or anakinra [71]. A 12-year-old girl suffered from NAIAD impaired left ventricular function and complete left bundle branch block, which proved to be rapidly reversible after initiation of high-dose methylprednisolone. Cytokine profiling revealed elevated IL-6, IL-10, and IL-18 and heterozygous mutation (c.3641C > G, p.Pro1214Arg) in the *NLRP1* gene [72]. This disease has been reported in individual cases, and the treatment measures are not unified.

### NLRC4-MAS (NLRC4-macrophage activation syndrome)

NLRC4-associated autoinflammatory disease (NLRC4-AID), including recurrent MAS and autoinflammation with infantile enterocolitis (AIFEC), is an autosomal dominant condition with GOF mutations of *NLRC4* [73]. Most of the mutations are located in the nucleotide-binding domain (NBD) and helicase domain 1 (HD1) of *NLRC4* and induce severe autoinflammation with high levels of IL-18 in the serum of the patients. NLRC4 is a cytosolic inflammasome activated in response to bacterial pathogen-associated molecular patterns (PAMPs). Once engaged, NLRC4 converts the precursor form of caspase-1 into a functional enzyme. In turn, caspase-1 cleaves pro-IL-1β and pro-IL-18 and induces pyroptosis or inflammatory cell death, releasing these cytokines into the extracellular space. The MAS phenotype is thought to be mediated by cytokine excess, particularly IL-1β and IL-18, with IFNγ also playing a role through its induction by IL-18. NLRC4-MAS patients have the extraordinary and chronic elevation of serum IL-18. Extremely elevated serum IL-18 concentrations are another biomarker in AIFEC/NLRC4-MAS patients that could be used to distinguish them from the primary hemophagocytic syndrome. Skin biopsy of a rash revealed neutrophil infiltrates and non-specific vasculitis lesions without fibrinoid necrosis.

Kawasaki et al. described a patient with clinical symptoms of severe CAPS whose disease was entirely responsive to IL-1 blockade and who was found to carry a high-frequency somatic *NLRC4* mutation. So, not all patients with NLRC4 inflammasomopathy develop MAS or enterocolitis [74]. A 60-year-old Brazilian female patient was evaluated for recurrent episodes of systemic inflammation from six months of age. Gene detection found a homozygous mutation in *NLRC4* (A160T), but inherited in a recessive fashion. This allele is significantly enriched in patients with ulcerative colitis: OR 2.546 (95% 1.778–3.644), *P* = *0.01305* [75]. Wang et al. reported a case of a late-onset autoinflammatory disease caused by a somatic *NLRC4* mutation in a small subset of leucocytes. A 69-year-old Chinese woman presented with recurrent rash and long-lasting fever episodes in her early 60 s. Skin biopsy suggested cutaneous vasculitis. Laboratory studies consistently showed elevated ESR (42–100 mm/hour), CRP (36–190 mg/L), and lactic dehydrogenase (LDH; 912–1800U/L). Whole-exome sequencing (WES) and whole-genome sequencing did not find any variants. Considering the late disease onset, they re-analyzed WES data for somatic mutation and identified a somatic missense variant c.1329C > G (p.His443Gln) in *NLRC4* mosaicism ratio of 2.61%. Allele-specific PCR and Sanger sequencing of PCR products also confirmed the presence of the somatic mutation. Therefore, the low ratio of somatic *NLRC4* mutation likely occurred as a late-onset event and clonally expanded mainly in the myeloid cells [76].

IL-1 blockade is effective in treating MAS-prone diseases but was not protective against the development of MAS. The effects of blocking IL-18 are unknown [77].

### PFIT (periodic fever, immunodeficiency, and thrombocytopenia)

Standing AS et al. reported a homozygous missense mutation in *WDR1* in two siblings causing periodic fevers with immunodeficiency and thrombocytopenia. They found impaired actin dynamics in patient immune cells. Patients had high serum levels of IL-18, without a corresponding increase in IL-18–binding protein or IL-1β, and their cells also secreted more IL-18 but not IL-1β in culture. Homozygous missense L153F/L293F mutation in the actin regulatory gene *WDR1* causes a new AID in humans, with periodic fevers, immunodeficiency, and intermittent thrombocytopenia (PFIT). Murine *WDR1*rd/rd mouse model induced by recessive *WDR1* mutations highlights a potentially crucial link between the actin cytoskeleton and autoinflammation. Although they cannot yet conclude that PFIT is driven purely by IL-18, the data suggest an essential role for this proinflammatory cytokine, driven by caspase-1 activation. A targeted IL-18 blockade might have been a more effective strategy to treat the disease. In the *WDR1*rd/rd mouse, transplant of bone marrow cells from wild-type mice corrected the autoinflammatory phenotype and macrothrombocytopenia. Given that severe autoinflammation coexists with immunodeficiency and thrombocytopenia in PFIT, hematopoietic stem cell transplantation (HSCT) may be the most appropriate treatment [78].

### APLAID (autoinflammation and PLCG2-associated antibody deficiency and immune dysregulation)

The auto-inflammation and phospholipase Cg2 (PLCg2)-associated antibody deficiency and immune dysregulation (APLAID) is an autosomal dominant/recessive autoinflammatory disease with a difficult diagnosis process in children. The APLAID patients presented with early-onset blistering skin lesions, posterior uveitis, IBD and recurrent sinopulmonary infections caused by a humoral defect but lacked autoantibodies and had no cold-induced urticaria. *PLCG2* encodes PLCg2, an enzyme responsible for ligand-mediated signaling in cells of the hematopoietic system, with a critical regulatory role in various immune and inflammatory pathways. This substitution may affect the normal Ca2 + ion fluctuation in cell and further studies about the role of Ca2 + ion and this syndrome is required. Zhou and his colleagues first reported it in 2012 in a father and his daughter who both suffered from early-onset recurrent skin inflammation and granulomata, nonspecific interstitial pneumonitis with respiratory bronchiolitis, arthralgia, eye inflammation, enterocolitis, cellulitis, and mild immunodeficiency. They caused by a GOF mutation S707Y in the *PLCG2* gene. They were refractory to treatment with NSAIDs and TNF inhibitors, and partially responsive to an IL-1 inhibitor. Their inflammatory manifestations were improved by high-dose corticosteroids, but the dosage and duration of treatment have been limited by side effects. Intravenous immunoglobulin (IVIG) was administered as treatment for hypogammaglobulinemia [79].

An 11-year-old girl with a history of repeated fever, myalgia, arthralgia, abdominal pain, and urticarial rash in the trunk and limbs was found a homozygote missense variant (c.579C > G, p. His193Gln) in exon 7 of the *PLCG2* gene. It suggests the probability that APLAID can act in an Autosomal Recessive manner in this family [80]. A 3-year-old boy with APLAID in Mexico experienced seizures and central nervous system vasculitis, which these symptom have not been previously described in APLAID. However as the number of patients with APLAID is small, it is possible that the overall spectrum of clinical manifestations has not been completely elucidated [81]. A 5-year-old boy presented with cutaneous erosions, severe scarring, depigmentation and contractures that were affecting major joints. Electron microscopy revealed a split in the sublaminadensa with the absence of anchoring fibrils, suggestive of dystrophic epidermolysis bullosa. Next-generation sequencing revealed a de novo heterozygous missense mutation in exon 22 of the *PLCG2*, which resulted in a substitution of serine by asparagine at codon 798 (p.Asp798Ser). The child was diagnosed with APLAID syndrome [82]. Therefore, APLAID has a variety of clinical manifestations. A 23-year-old Chinese Han man presented with recurrent fever for 18 years, vesiculopustular rashes for 9 years, chronic bronchitis, leukocytosis, increased C-reactive protein, immunodeficiency, and high serum IgE. Skin biopsy showed chronic inflammatory cell infiltration. A paternal heterozygous missense variant in exon 6 of the *PLCG2* gene p. I169V was identified. His vesiculopustular and IgE level responded to medium-dose corticosteroids. After withdrawal from steroids, he developed severe arthritis and large deteriorating ulceration resembling pyoderma gangrenosum on the left knee. Large dose corticosteroids were suboptimal. Then he received adalimumab with a satisfactory response for arthritis and skin lesion. But he got an immunodeficiency-associated lymphoproliferative disorder 2 months later [83]. Neves et al. described a new APLAID patient who presented with vesiculopustular rash in the 1st weeks of life, followed by IBD, posterior uveitis, recurrent chest infections, interstitial pneumonitis, and also had sensorineural deafness and cutis laxa. In this patient, a unique de novo heterozygous missense L848P mutation in the *PLCG2* gene was predicted to affect the PLCg2 structure. Her disease had been refractory to most treatments, including IL1 blockers and a trial with ruxolitinib had been attempted [84]. Nalda et al. described two unrelated patients suffering since the neonatal period from a complex disease mainly characterized by severe sterile inflammation, recurrent bacterial infections, and marked humoral immunodeficiency. Whole-exome sequencing detected a novel, de novo heterozygous *PLCG2* variant in each patient (p.Ala708Pro and p.Leu845_Leu848del). A clear enhanced PLCg2 activity for both variants was demonstrated by both ex vivo calcium responses of the patient’s B cells to IgM stimulation and in vitro assessment of PLCg2 activity. These data supported the APLAID diagnosis in both patients. An immunological evaluation revealed a severe decrease in immunoglobulins and B cells, especially class-switched memory B cells, with normal T and N. cell counts. The shreds of evidence here shown to expand APLAID diversity toward more severe phenotypes than previously reported, including dominantly inherited agammaglobulinemia, add novel data about its genetic basis [85]. Glucocorticoids, IVIG and biologics were clinically used to treat APLAID, but none of these patients had a complete recovery.

Vesiculopustular rashes, sinopulmonary infection and immunodeficiency are the most frequent symptoms of APLAID patients. The rarity and diversity of APLAID make it difficult to be diagnosed. Mutations at different loci of the *PLCG2* gene may expand the clinical phenotype and genotype of APLAID. There are few cases of the disease and a lack of experience in treatment. More research was needed to confirm the pathogenesis and management of APLAID.

## Conclusions

Clinical manifestations of SAIDs often occur sever, so early recognition and treatment are essential to reduce the incidence of complications and enhance the quality of life. With the availability of genetic sequencing technology, more patients and new autoinflammatory diseases will be diagnosed. There are numerous cases reported in the literature describing the successful use of anakinra and canakinumab to treat these IL-1 mediated disorders. However, IL-1 blocking treatments are not efficacious for all IL-1 mediated monogenic SAIDs, so other cytokines maybe play an essential role in the pathogenetic process in addition to IL-1. The molecular bases for cytokine amplification and the development of novel treatments are still to be discovered in most SAIDs. At the same time, it is also hoped that pediatricians, in the face of children with recurrent fever, recurrent rash, and poor effects of routine treatment, should be vigilant against SAIDs, and conduct gene testing and treatment in time.

## Data Availability

The data that support the findings of this study are available on request from the corresponding authors, MP Lu.

## Abbreviations

SAIDs: Systemic Autoinflammatory Diseases
CRP: C-reactive protein
ESR: Erythrocyte sedimentation rate
NSAIDs: Nonsteroidal anti-inflammatory drugs
MKD: Mevalonate kinase deficiency
AR: Autosomal recessive
LOF: Loss-of-function
MVK: Mevalonate kinase
HIDS: Hyperimmunoglobulinemia D and periodic fever syndrome
MA: Mevalonic aciduria
AA: AA amyloidosis
FMF: Familial Mediterranean fever
GOF: Gain-of-function;
TRAPS: TNF receptor associated periodic syndrome
AD: Autosomal dominant
TNFRSF1A: TNF receptor superfamily 1A
CNS: Central nervous system
SAA: Serum amyloid A
PAAND: Pyrin-Associated Autoinflammation with Neutrophilic Dermatosis
PAPA: Pyogenic arthritis, pyoderma gangrenosum (PG) and acne
PG: Pyoderma gangrenosum
PSTPIP1: Proline-serine-threonine phosphatase interacting protein 1
PYD: Protein pyrin domain
ASC: Apoptosis-associated speck-like protein containing a CARD
CAPS: Cryopyrin associated periodic syndromes
FCAS: Familial cold autoinflammatory syndrome
MWS: Muckle-Wells syndrome
NOMID: Neonatal-onset multisystem inflammatory disease
CINCA: Chronic infantile neurologic, cutaneous and articular syndrome;
DIRA: Deficiency of interleukin 1 receptor antagonist;
IL-1Ra: IL-1 receptor antagonist
MJS: Majeed syndrome
MRI: Magnetic resonance imaging
NAIAD: NLRP1-associated autoinflammation with arthritis and dyskeratosis syndrome
NLRC4-MAS: NLRC4-macrophage activation syndrome
AIFEC: Autoinflammation with infantile enterocolitis
PFIT: Periodic fever, immunodeficiency, and thrombocytopenia
APLAID: Autoinflammation and PLCG2-associated antibody deficiency and immune dysregulation
PLCg2: Phospholipase Cg2
